# A scoping review of the effect of chronic stretch training on sleep quality in people with sleep disorders

**DOI:** 10.1007/s00421-024-05541-z

**Published:** 2024-06-26

**Authors:** Alimoradi Mohammad, Hosseini Elham, Konrad Andreas

**Affiliations:** 1 Department of Sports Injuries and Corrective Exercises, Faculty of Sports Sciences, University of Shahid Bahonar Kerman, Kerman, Iran; 2 Institute of Human Movement Science, Sport and Health, Graz University, Mozartgasse 14, 8010 Graz, Austria

**Keywords:** Quality of sleep, Exercise therapy, Stretching exercises, Mental health

## Abstract

**Purpose:**

The widespread and health-detrimental sleep disorders have resulted in stretching exercises being investigated as a non-drug solution for enhanced sleep quality. However, a comprehensive understanding of the impact of stretching exercises on individuals with sleep disorders is lacking.

**Methods:**

This scoping review systematically maps the existing literature and identifies research gaps on the impact of stretching exercises on sleep quality in individuals with sleep disorders.

**Results:**

Sixteen eligible studies were included, where the weighted mean changes indicate a positive trend in sleep quality improvement, ranging from trivial to very large magnitudes. However, concerning the individual study results only 5 out of 16 studies reported significant improvements. Notable enhancements include a small 1.22% overall sleep quality improvement, a large 6.51% reduction in insomnia severity, a large 8.88% increase in sleep efficiency, a moderate 4.36% decrease in sleep onset latency, a large 8.27% decrease in wake after sleep onset, and a very large 14.70% improvement in total sleep time. Trivial changes are noted in sleep duration (0.58%), sleep disturbance reduction (0.07%), and daytime dysfunction reduction (0.19%). Likely mechanisms for the improvement of sleep include autonomic nervous system modulation, muscle tension relief, cortisol regulation, enhanced blood circulation, and psychological benefits such as stress reduction and mood enhancement.

**Conclusion:**

There is little evidence that stretching exercises positively impact sleep quality in individuals with sleep disorders. Additionally, further research is vital for designing optimal protocols, understanding of the long-term effects, and clarification of the mechanisms.

## Introduction

Sufficient sleep plays a vital role in promoting both physical and mental health, as well as enhancing overall feelings of wellness (Chen et al. 2006). Insufficient sleep can result in heightened fatigue and excessive daytime sleepiness (Bliwise 1996). Moreover, sleep-related difficulties have detrimental effects on both mental and physical health, as well as on the overall quality of life and the financial burden of healthcare (Kripke et al. 2002; Simon and VonKorff 1997). For example, research has demonstrated that prolonged sleep disturbances are associated with enduring health consequences, encompassing conditions such as high blood pressure, cardiovascular diseases, stroke, depression, and even death (Malhotra 2008). Another investigations indicated that sleep disorders can precipitate a range of health issues, including sleep-disordered breathing, immune system disorders, dementia, pain, and depression (Ancoli-Israel et al. 1997; Tan et al. 2019; Roberts and Drummond 2016; Jindal and Thase 2004; Guarnieri 2019). Moreover, solely within the United States, the expenditure for diagnosing and treating sleep-related issues (including medical treatment) amounted to roughly $12.4 billion in 2015 (Watson 2016). Approximately 30% of the general population encounters sleep disorders, with 10% experiencing disruptions in sleep patterns and daytime dysfunction (National Institutes of Health 2005). Research has indicated that the incidence of sleep disorders in adults ranges from 9 to 12%, while in older adults it reaches 20% to 30% (Irwin et al. 2006; Gu et al. 2010). Historically, the management of sleep disorders has predominantly relied on pharmaceutical interventions, encompassing the use of medication such as benzodiazepines (Chen et al. 2016). However, long-term use of these medications can induce potential adverse effects, including dependency and drug resistance, while concurrently contributing to cognitive decline in individuals with sleep disorders (Zee et al. 2023; Jaseja et al. 2021).

Hence, exercise serves as a non-pharmacological intervention for sleep-related issues, being easily accessible and more cost-effective than alternative non-pharmacological treatments (Youngstedt et al. 1997; Driver and Taylor 2000; Youngstedt 2005). Recent randomized controlled trials have substantiated the beneficial impact of physical exercise on various aspects of sleep, including sleep quality, sleep latency, the total duration of sleep, sleep efficiency, and insomnia severity (Passos et al. 2012; Reid et al. 2010; Hartescu et al. 2015). However, while there is increasing evidence supporting the positive effects of exercise on sleep, further investigation is needed to examine the specific effects of different exercise protocols on sleep quality in individuals with sleep disorders.

Stretching exercises are a widely practiced form of physical activity, with the goal to increase the range of motion of a joint acutely as well as chronically (Alimoradi et al. 2023; Behm et al. 2023; Konrad et al. 2023a). If the goal is to increase the range of motion, in the long term, stretching is commonly recommended as a stand-alone activity to decrease muscle stiffness as well as muscle–tendon unit stiffness, alleviate muscle tension, and promote relaxation (Nakamura et al. 2024; Small et al. 2008; Takeuchi et al. 2023a, 2023b). While the effects of stretching on muscle function and structure have been extensively studied, its potential impact on sleep quality remains an area that requires further exploration. Understanding the relationship between stretching exercises and sleep outcomes could provide valuable insights into the non-pharmacological management of sleep disorders.

Therefore, the purpose of this scoping review is to give an overview of the existing literature on the effect of stretching exercise protocols on sleep quality in a variety of individuals with sleep disorders. By synthesizing and analyzing the available research, this review aims to provide a comprehensive overview of the current state of knowledge, identify research gaps, and highlight areas for future investigation. The findings of this review will have implications for the development of stretch exercise-based interventions targeting sleep improvement in individuals with sleep disorders, ultimately contributing to the enhancement of overall health and well-being.

## Definition and measurement of sleep quality

### Sleep quality

Sleep quality refers to the subjective perception of the depth, continuity, and restfulness of sleep experienced during the night. It encompasses various aspects such as sleep duration, sleep latency (time taken to fall asleep), sleep efficiency (percentage of time spent asleep while in bed), sleep disturbance, and overall satisfaction with sleep. Optimal sleep quality is characterized by uninterrupted, restorative sleep, leading to the individual feeling refreshed upon waking (Fabbri et al. 2021; Krystal and Edinger 2008).

### Measurement of sleep quality

Measuring sleep quality typically involves subjective self-report measures, objective assessments, or a combination of both. Two widely used tools for assessing sleep quality are polysomnography (PSG) and the Pittsburgh Sleep Quality Index (PSQI) (Buysse et al. 1989; Fabbri et al. 2021).

### Polysomnography (PSG)

PSG is considered the gold standard for objective sleep assessment. It involves monitoring various physiological parameters during sleep, including brain waves (electroencephalography or EEG), eye movements (electrooculography or EOG), muscle activity (electromyography or EMG), heart rate, respiratory effort, and oxygen saturation. PSG provides detailed information about sleep architecture, including the distribution of sleep stages (such as rapid eye movement (REM) sleep and non-REM sleep), sleep continuity, and the presence of sleep disorders such as sleep apnea or periodic limb movement disorder (Rundo and Downey 2019; Dredla et al. 2023).

### Pittsburgh sleep quality index (PSQI)

The PSQI is a widely used self-report questionnaire designed to assess subjective sleep quality over a one-month period. It consists of 19 items that generate seven component scores: subjective sleep quality, sleep latency, sleep duration, habitual sleep efficiency, sleep disturbance, use of sleep medication, and daytime dysfunction. Each component score ranges from 0 to 3, with higher scores indicating poorer sleep quality. The sum of the component scores yields a global PSQI score ranging from 0 to 21, with scores greater than 5 indicating poor sleep quality. The PSQI provides a comprehensive overview of various dimensions of sleep quality and is suitable for both clinical and research settings, due to its simplicity and ease of administration (Buysse et al. 1989).

### Role of PSG and the PSQI in sleep research

Both PSG and the PSQI play complementary roles in sleep research. PSG offers detailed objective data on sleep architecture and physiology, allowing for precise diagnosis and characterization of sleep disorders. However, PSG may not always capture subjective experiences or factors influencing sleep quality, such as environmental conditions or psychological factors. In contrast, the PSQI provides valuable insights into individuals’ subjective perception of sleep quality, including subjective sleep disturbances and daytime functioning. The PSQI is a useful tool for assessing sleep quality in large-scale studies, clinical settings, and epidemiological research (Fabbri et al. 2021; De Fazio et al. 2022).

In conclusion, sleep quality is a multifaceted construct that is crucial for overall health and well-being. Objective measures such as PSG and subjective assessments such as the PSQI offer complementary approaches for evaluating sleep quality, providing valuable insights into individuals’ sleep patterns, and potential interventions for improving sleep health.

## Materials and methods

**Fig. 1:**
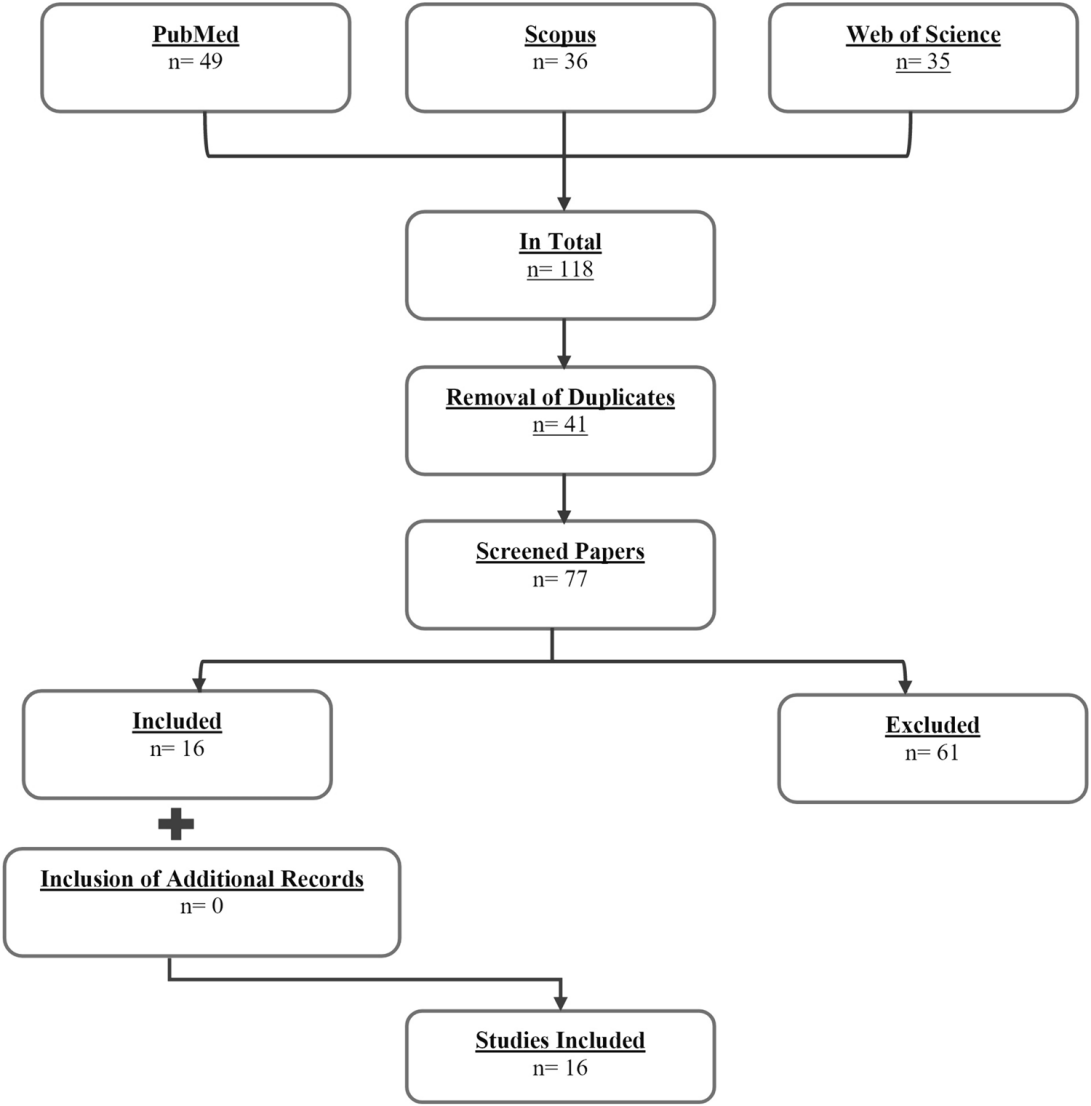
PRISMA flowchart of the systematic screening process (Preferred Reporting Items for Systematic Reviews and Meta-analyses)

This review adheres to the recommendations provided by Munn et al. (2018) for conducting scoping reviews, with the primary objectives being the identification of available evidence and the identification of gaps in knowledge. This review encompasses scientific studies that have examined the impact of various forms of stretching exercises on the quality of sleep. We focused exclusively on studies that examined the effects of stretching alone, without any combined treatments involving stretching and other interventions (e.g., foam rolling). This approach allowed us to isolate the effects of stretching on sleep quality. In addition, we included participants from diverse backgrounds, while also encompassing individuals with various types of diseases. By including participants with different medical conditions, we aimed to explore the potential benefits of stretching on sleep quality across a wide range of populations. Furthermore, we specifically considered the long-term effects of stretching (i.e., chronic studies and training studies) and excluded acute studies from the review. The electronic literature search was conducted across three distinct databases (PubMed, Scopus, and Web of Science), from their point of inception to April 2023. To identify pertinent studies concerning stretching in the context of sleep disorders, we employed a comprehensive search strategy encompassing various terms associated with sleep disorders, including insomnia, 'sleep problem,' 'sleep disorder,' 'sleep complaints,' 'sleep disturbance,' 'sleep quality,' 'dyssomnia,' 'extrinsic sleep disorder,' and 'sleep initiation and maintenance disorder.' Additionally, we incorporated the term 'stretch*' (with an AND binder) to ensure the inclusion of relevant literature on stretching interventions within this domain. Only studies written in the English language were found in the search, leading to the identification of a total of 118 studies throughout the three databases. Following the exclusion of duplicate studies (*n* = 41), the remaining articles were individually assessed by two researchers (M.A. and E.H.), based on their titles as well as abstracts, to determine the inclusion of the studies in this review. After the independent screening process, the researchers compared their results and deliberated on any discrepancies. If there was no agreement, a third person was consulted (A.K.). Out of the 77 initially screened studies, 16 studies were deemed suitable, according to the inclusion criteria. No additional eligible studies were found through a supplementary search of the references and citations via Scopus in the 16 already included papers. A comprehensive depiction of the search methodology can be found in Fig. 1. The subsequent sections present the weighted mean percentage changes (pre to post) and the corresponding 95% confidence intervals (CIs) for the effects of stretching training on sleep outcomes (i.e., sleep quality, insomnia severity, sleep efficiency, sleep onset latency, REM latency, wake after sleep onset). Furthermore, in line with previous recommendations (Behm et al. 2015; Konrad et al. 2023b), we categorized the calculated percentage weighted mean changes in the parameters into different magnitudes: less than 0.5% as trivial, 0.5% to less than 2% as small, 2% to less than 5% as moderate, 5% to less than 10% as large, and greater than 10% as very large.

## Overview of the eligible studies

Sixteen studies of the effects of stretching training on sleep quality were included in this review. These studies included a total of 1281 participants, with an average age of 58 ± 4 years. Detailed information about the population, the specific stretching exercises employed, and the outcomes for all the measured parameters, as well as the mean percentage changes (pre-test to post-test) of the stretching training on sleep outcomes in the included studies, can be found in Tables 1 and 2. Among the 16 studies examined, 13 studies included sleep quality outcomes, such as sleep quality, duration, efficiency, and insomnia severity (refer to Table 1). In addition, a further three studies investigated leg cramps during sleep, as outlined in Table 2, which can be recognized as a significant factor influencing sleep quality.

**Table 1:** Summary of studies which have investigated the effect of stretching training on sleep components

Study	Subjects	Stretching intervention		Main outcome measured	Percentage change (pre to post) of the main outcome
		Muscle (group)	Type/duration		
D’Aurea et al. (2018)	28 participants with insomnia complaints Age: 43.4 ± 2.4 years	Upper and lower limbs	16 weeks: 5-min walk, 45-min stretching (8–10 stretches/region)	(Wrist actigraphy)	
				Insomnia severity	↓ 43.3%^a^
				Sleep onset latency	↓ 20.0%^a^
				Total sleep time	↑ 5.9%^a^
				Wake after sleep onset	↓ 23.5%^a^
				Sleep efficiency	↑ 5.6%^a^
				(Polysomnography)	
				Sleep onset latency	↑ 20.0%^b^
				Total sleep time	↑ 11.7%^b^
				Wake after sleep onset	↓ 22.2%^b^
				Sleep efficiency	↑ 5.7%^b^
				Rapid eye movement latency	↓ 30.6%^b^
				(PSQI)	
				Global score	↑ 32.5%^a^
				Sleep duration	↑ 37.2%^a^
				Sleep latency	↓56.3%^a^
				Sleep efficiency	↑ 20.4%^a^
Calandre et al. (2009)	81 male and female participants with fibromyalgia Age: 50.0 ± 8.2 years	Main body: cervical, upper, lower, and trunk areas	6 weeks: 3 sessions × 60 min per week	PSQI total score	↑ 7.8%^b^
				Subjective sleep quality	↓ 15.1%^b^
				Sleep latency	↓ 11.1%^b^
				Sleep duration	↓ 12.2%^b^
				Sleep efficiency	↓ 8.2%^b^
				Sleep disturbance	↓ 0.5%^b^
				Sleep medication	↔
				Daytime dysfunction	↓ 14.2%^b^
(Huberty et al. 2020)	90 females with experience of stillbirth Age: NR	NR	12 weeks online stretching / toning exercise group 60 min per week (1–2 sets of 20–45 s or 10–15 reps)	Sleep quality	Stretch group = ↓ 10.1%^b^
Imboden et al. (2021)	43 patients with moderate-to-severe depression Age: 39.9 ± 11.4 years	Whole body	6 weeks: 3 times per week	PSQI score	↑ 30.3%^a^
				(Polysomnography)	
				Sleep efficiency	↑ 1.9%^b^
				Total sleep time	↑ 0.4%^b^
				Sleep onset latency	↓ 22.2%^b^
				Wake after sleep onset	↑ 29.1%^b^
				Rapid eye movement latency	↑ 20.5%^b^
				Awakenings	↑ 35.4%^a^
Ratcliff et al. (2016)	163 females with breast cancer Age: 51.8 ± 1.3 years	Whole body	6 weeks: 3 sessions × 60 min per week	Sleep quality	Stretch group = ↑ 2.3%^b^
Chandwani et al. (2014)	163 women with breast cancer Age: 51.8 ± 1.3 years	Whole body	6 weeks: 3 sessions × 60 min per week	Sleep quality	Stretch group = ↑ 2.3%^c^
Afonso et al. (2012)	44 postmenopausal women Age: between 50 and 65 years	Whole body	12 weeks passive stretching	Insomnia severity	Stretch group = ↓ 32.5%^b^
Choudhary et al. (2019)	144 older adults Age: NR	Whole body	12 weeks conventional stretching; 3 times × 60 min per week	Insomnia severity	Stretch group = NR%^c^
Bullock et al. (2020)	61 older adults Age: 71.9 ± 5.7 years	Whole body	12 weeks: 30 min, with each stretch held for 30–40 s	Sleep duration	↓ 4.2%^b^
				Sleep efficiency	↑ 6.2%^b^
				Global PSQI score	↑ 9.6%^b^
				Subjective sleep quality	↑ 12.5%^b^
				Sleep latency	↓ 46.1%^b^
				Habitual sleep efficiency	↓ 16.6%^b^
				Sleep disturbance	↓ 13.3%^b^
				Use of sleeping medication	↓ 14.2%^b^
				Daytime dysfunction	↔
Yu et al. (2022)	37 middle aged and older adults Age: 63.4 ± 5.5 years	Chest, back, and lower limbs	12 weeks: once a week (75 min each session)	Sleep quality	↓ 5.45%^b^
Gouw et al. (2018)	24 pain-free males and females with sleep bruxism Age: 34.6 ± 9.1 years	Masticatory muscle	Static stretching with a device for a total of 10 days	Sleep bruxism episodes	↑18.5%^b^
				Sleep bruxism bursts	↑ 22.8%^a^
				Sleep duration	↑ 3.9%^b^
				Sleep quality	↑7.4%^b^
Kline et al. (2012)	43 sedentary and overweight/obese adults with at least moderate-severity untreated OSA (apnea–hypopnea index ≥ 15) Age: 46.7 ± 1.7 years	Whole body	12 weeks: 10–15 stretches held for 15–30 s twice a week	Daytime sleepiness	↑ 8.2%^b^
Wang et al. (2022)	60 patients with Parkinson’s disease Age: 67.6 ± 4.3 years	Whole body	24 weeks: 60 min per week	Parkinson’s Disease Sleep Scale	↑ 3.7%^a^

↑ = indicates increase in outcome

↓ = indicates deterioration in outcome

↔  = no change

*NR* not reported

*PSQI* Pittsburgh Sleep Quality Index

^a^ significant change between pre-test and post-test

^b^ non-significant change between pre-test and post-test

^c^ the level of significance was not reported

**Table 2:** Summary of studies which have investigated the effect of stretching exercises on leg cramps during sleep

Study	Subjects	Stretching intervention		Main outcome measured	Percentage change (pre to post) of the main outcome
		Muscle (group)	Type/duration		
Hallegraeff & de Greef (2020)	29 female frail older adults Age: 84.9 ± 3.8 years	NR	3 stretch exercises 1 × 20 s each for 6 weeks	NLC frequency	↓ 28.5%^a^
				NLC intensity	↓ 14.7%^a^
Hallegraeff et al. (2012)	80 male and female older adults Age: 70.0 ± 6.0 years	Hamstrings and calves	2 dynamic stretch exercises 3 × 10 s each for 6 weeks	NLC frequency	↓ 58.8%^a^
				NLC severity	↓ 18%^a^
Coppin et al. (2005)	191 patients prescribed quinine for night cramps Age: 74.7 ± 0.6	Calf and foot muscles	6 weeks: 3 × 20 s each (after 10 s, participants took rest for 5 s then repeated the stretch for 10 s)	Frequency of night cramps	NR%^b^
				Severity of night cramps	NR%^b^

↓ = indicates deterioration in outcome

*NLC* nocturnal leg cramps

*NR* not reported

^a^ significant change between pre-test and post-test

^b^ non-significant change between pre-test and post-test

### Sleep quality outcomes

The 13 included studies encompassed a diverse range of populations, including individuals with insomnia complaints, fibromyalgia, stillbirth experience, depression, breast cancer, postmenopausal women, older adults, and those with sleep bruxism, Parkinson’s disease, or untreated obstructive sleep apnea (OSA). The stretching interventions varied widely in terms of duration, frequency, and targeted muscle groups (see Table 1). Overall, out of the 13 studies, three reported significant improvements (at least in one parameter) (D’Aurea et al. 2018; Imboden et al. 2021; Wang et al. 2022). The remaining 10 studies reported no significant change in any parameter (Afonso et al. 2012; Bullock et al. 2020; Calandre et al. 2009; Chandwani et al. 2014; Choudhary et al. 2019; Gouw et al. 2018; Huberty et al. 2020; Kline et al. 2012; Ratcliff et al. 2016; Yu et al. 2022).

The mean effect of the stretching training on sleep quality showed a small improvement of 1.22% (95% CI 1.14–1.30%), based on data from 6 studies. For insomnia severity, there was a large mean improvement of 6.51% (95% CI 5.77–7.25%), combining findings from three studies. Sleep efficiency demonstrated a large 8.88% improvement (95% CI 8.48–8.98%), with data synthesized from four studies. Sleep onset latency decreased by 4.36% (95% CI 3.75–4.45%), with a moderate magnitude, across four studies. Total sleep time increased by 14.70% (95% CI 14.33–15.07%), with a very large magnitude, aggregating results from two studies. For sleep duration, a 0.58% improvement (95% CI 0.57–0.59%) was shown, with a trivial magnitude, based on data from three studies. Sleep disturbance decreased by 0.07% (95% CI 0.007–0.14%), with a trivial magnitude, drawn from two studies. Use of sleeping medication did not change (0.04%; 95% CI − 0.03–0.13%) based on data from two studies. Daytime dysfunction decreased by 0.19% (95% CI 0.14–0.24%), with a trivial magnitude, as reported in the data from two studies. Lastly, wake after sleep onset decreased by 8.27% (95% CI 7.80–8.74%), with a large magnitude, drawing from two studies. Since sleep bruxism episodes, sleep bruxism bursts, and daytime sleepiness were assessed in one study only, no weighted mean changes could be assessed. Overall, the weighted mean changes show a positive trend for improving sleep in the various parameters, with a magnitude between trivial to very large. While the weighted mean changes indicate an overall positive trend, it is also important to note that the CIs show one direction, suggesting improvement in sleep parameters. However, the width of the CIs indicates some variability or uncertainty in the precise magnitude of these improvements. In addition, it is worth mentioning that the individual studies reported either significant improvements in the various parameters or no changes. Though, considering the individual study results only 3 out of 13 studies reported significant improvements in sleep quality parameters.

D’Aurea et al. (2018) conducted a 16-week intervention with participants experiencing insomnia complaints, incorporating upper and lower limb stretching. This study reported a significant decrease in insomnia severity and improvements in sleep quality and sleep-related parameters (D’Aurea et al. 2018). Huberty et al. (2020) found that a stretching protocol employed for 12 weeks showed a non-significant reduction in sleep quality among women who had experienced stillbirth. Imboden et al. (2021) explored the impact of whole-body stretching on sleep parameters in patients with moderate-to-severe depression. The 6-week intervention resulted in no change of the most objectively measured data from PSG, including sleep efficiency, total sleep time, sleep onset latency, wake after sleep onset, and REM latency, over the intervention period, without any significant effects of time or time by group. However, there was a significant reduction in the number of awakenings (ES = 0.41), as well as significantly improved sleep quality (ES = 0.16) (Imboden et al. 2021). Two studies by Ratcliff et al. (2016) and Chandwani et al. (2014) focused on sleep quality in females with breast cancer. Although stretching interventions showed positive trends in sleep quality after 6 weeks, the results did not reach statistical significance (Chandwani et al. 2014; Ratcliff et al. 2016). Afonso et al. (2012) reported similar positive outcomes in postmenopausal women undergoing 12 weeks of passive stretching. Following the treatment, there was a 32.5% decrease in insomnia severity, although it was not statistically significant compared to the control group. Calandre et al. (2009) investigated the effects of stretching on sleep quality and sleep-related parameters in individuals with fibromyalgia. After a 6-week intervention targeting the cervical, upper, lower, and trunk areas, non-significant improvements were observed in various parameters of the PSQI, including total PSQI (ES = 0.28), reduced sleep latency (ES = 0.28), duration (ES = 0.25), efficiency (ES = 0.15), disturbance (ES = 0.04), and daytime dysfunction (ES = 0.34). Three studies evaluated the effects of different stretching exercises in middle-aged and older adults (Bullock et al. 2020; Choudhary et al. 2019; Yu et al. 2022). The results indicated that the stretching interventions had a non-significant impact on reducing insomnia severity, sleep duration, sleep latency, habitual sleep efficiency, sleep disturbance, and the use of sleeping medication, after a period of 12 weeks. (Bullock et al. 2020; Choudhary et al. 2019; Yu et al. 2022). Bullock et al. (2020) investigated poor sleep in individuals undergoing a 12-week stretching protocol. The results showed non-significant improvements in sleep parameters such as efficiency (11.2%), PSQI score (13.5%), and duration (8.3%). In addition, non-significant reductions in latency (47.3%), sleep disturbance (11.1%), and medication use (37.5%) were observed. Similarly, Gouw et al. (2018) reported that static stretching for 10 days had no impact on sleep duration or sleep quality in individuals with sleep bruxism. Moreover, there was a non-significant increase in sleep bruxism episodes. Furthermore, the authors reported a significant increase in sleep bruxism bursts (Gouw et al. 2018). Ultimately, Kline et al. (2012) and Wang et al. (2022) investigated stretching exercises in sedentary and overweight individuals, and also Parkinson’s patients. In the Kline et al. (2012) study, daytime sleepiness was measured by the Epworth Sleepiness Scale, and the results showed a non-significant difference (ES =  − 0.67) after the 12-week stretching intervention, from baseline to post-intervention. Wang et al. (2022) evaluated sleep condition in Parkinson’s patients with the Parkinson’s Disease Sleep Scale (PDSS) and the stretching exercises resulted in a significant change (ES = 0.15) in PDSS scores after 24 weeks.

In conclusion, ten out of 13 studies have indicated non-significant improvements in sleep quality. However, the analysis of the weighted mean changes showed trivial to very large changes for improvements in the various sleep outcomes. Though, the diversity of the populations, intervention durations, and methodologies underscores the complexity of addressing sleep-related parameters through stretching interventions. Further research should provide a better understanding of the factors influencing the efficacy of these interventions on sleep quality.

### Leg cramps during sleep

Leg cramps during sleep, which are a factor influencing sleep quality, were assessed in three studies (Coppin et al. 2005; Hallegraeff and de Greef 2020; Hallegraeff et al. 2012). The interventions targeted frail older adults, older adults, and patients prescribed quinine for night cramps. Two out of the three studies reported that the stretching exercises resulted in a significant reduction of nocturnal leg cramps during sleep (see Table 2). Small reductions in nocturnal leg cramp frequency and intensity were observed across these studies: 1.72% (95% CI 1.32–2.13) for nocturnal leg cramp frequency and 1.19% (95% CI 0.90–1.48%) for nocturnal leg cramp severity. Hallegraeff and de Greef (2020) conducted a 6-week intervention with frail older adults, resulting in a significant decrease in nocturnal leg cramp frequency (from 3.5 cramps per night at baseline to 2.5 after intervention; 95% CI 0.3–1.5) and intensity of pain (6.1 at baseline to 5.2 after intervention; 95% CI 0.4–1.3). In addition, the results between the experimental and control groups for both the frequency (p = 0.04) and intensity (p = 0.01) of nocturnal leg cramps were significant (Hallegraeff & de Greef 2020). Another study by Hallegraeff et al. (2012) involved dynamic stretching exercises in older adults for 6 weeks, demonstrating a significant reduction in both leg cramp frequency (mean difference 1.2 cramps per night; 95% CI 0.6–1.8) and severity (mean difference 1.3 cm; 95% CI 0.9–1.7), compared to the control group. Coppin et al. (2005) implemented a 6-week stretching intervention in patients prescribed quinine for night cramps and found that this was not effective in decreasing the frequency (mean difference 1.95; 95% CI − 3.01–6.90) or intensity (mean difference 0.02; 95% CI − 0.35–0.38) of leg cramps during sleep.

In conclusion, interventions targeting nocturnal leg cramps demonstrate potential, with a small magnitude of change, in improving both the frequency and severity of nocturnal leg cramps, particularly in frail older adults and older adults. However, the efficacy of these interventions can vary, as evidenced by the mixed outcomes observed in patients prescribed quinine for night cramps. These findings highlight the need for further research to refine and tailor interventions for specific populations, ultimately contributing to enhanced sleep quality and overall well-being.

### Possible mechanisms for changes in sleep parameters

Understanding the underlying mechanisms by which stretching exercise protocols might influence sleep quality is crucial for developing effective interventions for individuals with sleep disorders. Investigating these mechanisms could provide insights into the physiological and psychological pathways through which stretching exercises can benefit sleep. Previous research has established a link between physical exercise and improved sleep quality (Bonardi et al. 2016; Kelley and Kelley 2017). Studies have suggested that exercise, in general, can promote better sleep by influencing various physiological and psychological factors. However, the specific mechanisms by which stretching exercises impact sleep quality in individuals with sleep disorders remain less explored.

**Fig. 2:**
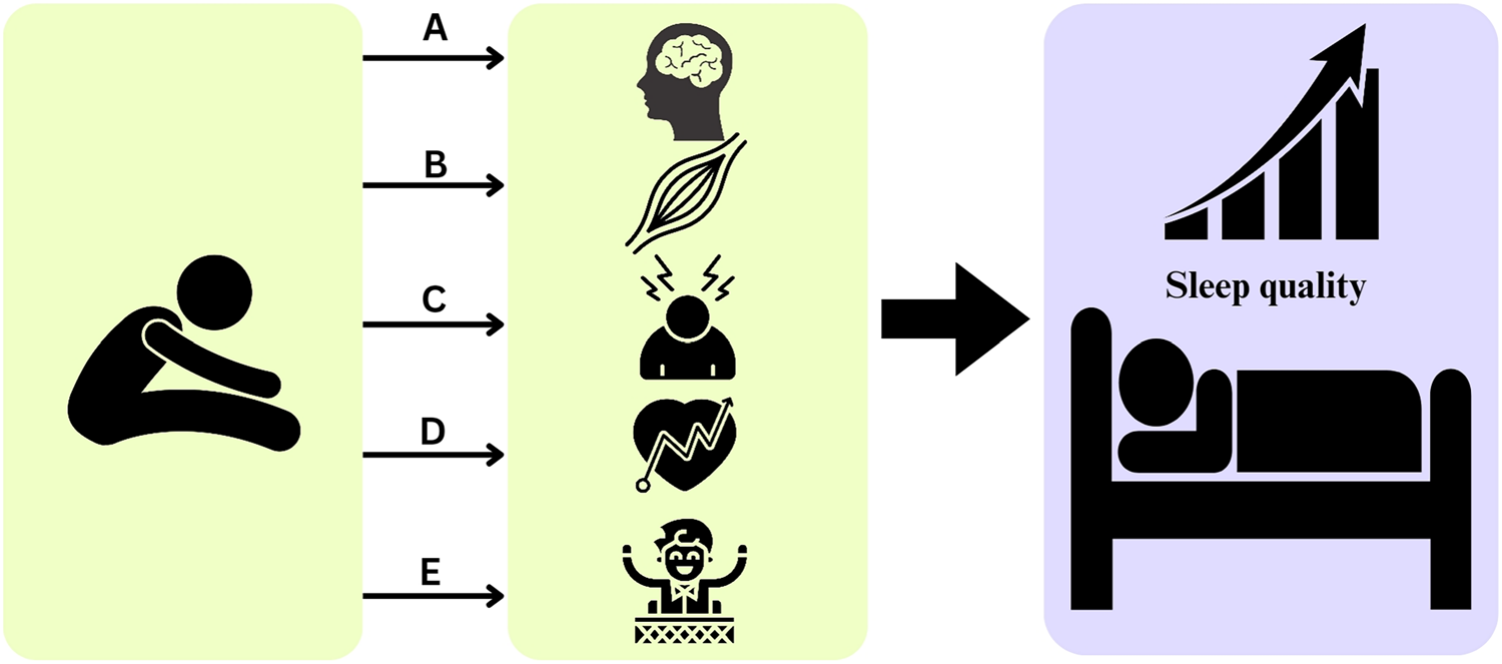
Stretching exercises can impact sleep quality by **A** modulating the autonomic nervous system, **B** promoting muscle relaxation, **C** regulating stress hormone levels, **D** enhancing blood circulation, and **E** providing psychological benefits such as stress reduction and mood enhancement

Stretching exercises can influence the autonomic nervous system (see Fig. 2), which plays a crucial role in sleep regulation (Imagawa et al. 2023). Activation of the parasympathetic branch and suppression of the sympathetic branch of the autonomic nervous system during and after stretching exercises can induce a state of relaxation and calmness, facilitating the transition into sleep (Sakurai et al. 2024; Imagawa et al. 2023). The stimulation of the parasympathetic nervous system, in particular, can promote sleep initiation and maintenance (Kim et al. 2022). Stretching exercises alleviate muscle tension and promote relaxation, thus aiding in better sleep quality (D’Aurea et al. 2018). By targeting specific muscle groups, stretching relieves tightness and discomfort often associated with sleep disturbance (D’Aurea et al. 2018). Improved circulation, the release of beta-endorphin, and the establishment of a bedtime routine contribute to relaxation and signal the body for sleep (Çoban et al. 2023; Franco et al. 2019). Overall, incorporating stretching into one’s pre-bedtime routine can significantly enhance sleep quality and overall well-being. Stretching exercises can also modulate the release of stress hormones, such as cortisol (Corey et al. 2014). Cortisol follows a diurnal rhythm, with levels typically higher in the morning and lower in the evening (Van Cauter et al. 1996). Regular stretching in the evening can help regulate cortisol secretion, promoting a natural decline in cortisol levels closer to bedtime. This regulation contributes to improved sleep onset and maintenance (Corey et al. 2014; Imboden et al. 2021). Stretching exercises play a vital role in enhancing blood circulation to the muscles being stretched, thereby improving the delivery of oxygen and nutrients. Consequently, chronic stretching practices improve vascular function and flexibility, leading to better overall circulation (Kato et al. 2020). This improved blood flow acts as a physiological signal to the body, indicating a state of relaxation and aiding in recovery (Hotta et al. 2013). By ensuring optimal oxygenation and nutrient supply to the body's tissues, stretching indirectly supports sleep quality. This improved circulation not only helps alleviate muscle tension but also promotes overall physical relaxation, contributing to a more conducive environment for restful sleep (Ujikawa and Koga 2020; Baklouti et al. 2023). Therefore, incorporating stretching exercises into one's evening routine can have a positive impact on both physical and mental well-being, ultimately leading to enhanced sleep quality and overall health. Additionally, stretching exercises offer psychological benefits, including stress reduction, anxiety relief, and mood enhancement. Stretching promotes calmness and relaxation, assisting individuals with sleep disorders in unwinding before bedtime (Choudhary et al. 2019; Ratcliff et al. 2016). This reduction in psychological distress and enhancement in mood, as suggested by Geiker et al. (2018), create a more conducive sleep environment, leading to improved sleep quality. Therefore, integrating stretching into an evening routine not only addresses physical tension but also fosters mental well-being, ultimately promoting better overall sleep. These physiological mechanisms interact synergistically to enhance sleep quality. By combining the effects of autonomic nervous system modulation, muscle tension reduction, stress hormone regulation, enhanced blood circulation, and psychological benefits, stretching exercises create an optimal physiological state conducive to restful sleep.

### Heterogeneity in the intervention characteristics

While the weighted mean changes showed a positive trend in sleep quality improvement, it is crucial to acknowledge the considerable heterogeneity in the intervention characteristics. The diversity in stretching types, duration, frequency, and targeted muscle groups may have contributed to the variability in the reported outcomes. Future research should aim to identify optimal parameters for stretching interventions tailored to specific sleep-related issues, thus facilitating the development of more targeted and effective non-pharmacological interventions.

### Leg cramps during sleep as a mediator of sleep quality

The investigation of leg cramps during sleep as a mediator of sleep quality revealed small reductions in both the frequency and intensity of nocturnal leg cramps following stretching interventions. This suggests that the positive impact of stretching on sleep quality may, in part, be mediated through a reduction in nocturnal leg cramps during sleep. While the exact mechanisms underlying this relationship warrant further exploration, the observed outcomes highlight an additional dimension to the multifaceted benefits of stretching exercises in sleep improvement.

### Methodological considerations and research gaps

It is crucial to acknowledge certain methodological limitations across the included studies. The heterogeneity in outcome measures, the lack of standardized stretching exercises, and the variations in sample pose challenges when drawing definitive conclusions. In addition, the majority of the studies predominantly relied on self-reported measures of sleep quality, introducing potential bias. Future research should incorporate objective measures such as actigraphy and PSG, to enhance the robustness of the findings (Faerman et al. 2020; McCall and McCall 2012). Furthermore, the duration of the follow-up assessments in the included studies varied, ranging from 6 to 24 weeks (Afonso et al. 2012; Bullock et al. 2020; Calandre et al. 2009; Chandwani et al. 2014; Choudhary et al. 2019; Coppin et al. 2005; D’Aurea et al. 2018; Hallegraeff and de Greef 2020; Hallegraeff et al. 2012; Huberty et al. 2020; Imboden et al. 2021; Kline et al. 2012; Ratcliff et al. 2016; Wang et al. 2022; Yu et al. 2022). Longer-term investigations are warranted to ascertain the sustainability and long-lasting effects of stretching interventions on sleep quality. In addition, the current literature predominantly focuses on the adult and older adult populations, leaving a notable gap in our understanding of the effects of stretching exercises on sleep in younger individuals and children.

### Clinical implications and recommendations

The positive trend of improvement has potential implications for the development of non-pharmacological interventions for individuals with sleep disorders. Stretching exercises offer a cost-effective, accessible, and low-risk alternative to pharmacological interventions. Clinicians and healthcare providers could consider incorporating tailored stretching exercises into comprehensive sleep improvement strategies. However, the specific prescription of stretching exercises should be individualized, based on the underlying sleep-related issue, and consultation with healthcare professionals is advised.

## Conclusion and future directions

In conclusion, this scoping review provides a comprehensive overview of the current state of knowledge regarding the impact of stretching exercise protocols on sleep quality. A positive trend of improvement across heterogeneous populations indicates the potential use of stretching exercises as an adjunctive or stand-alone intervention for individuals with sleep-related issues. However, it has to be noted that the majority of the individual studies (10 out of 13) reported non-significant improvements in sleep quality parameters. Future research should prioritize standardization of stretching exercises, explore the underlying mechanisms linking stretching exercises to improved sleep, and address research gaps in specific populations and age groups.

## Data Availability

The original contributions presented in the study are included in the article. Further inquiries can be directed to the corresponding author.

## Abbreviations

CI: Confidence interval
PSG: Polysomnography
EEG: Electroencephalography
EOG: Electrooculography
EMG: Electromyography
REM: Rapid eye movement
PSQI: Pittsburgh Sleep Quality Index
OSA: Obstructive sleep apnea
ES: Effect size
PDSS: Parkinson’s Disease Sleep Scale
